# Fibroblast growth factor 12 is expressed in spiral and vestibular ganglia and necessary for auditory and equilibrium function

**DOI:** 10.1038/s41598-018-28618-0

**Published:** 2018-07-31

**Authors:** Yukiko Hanada, Yukiko Nakamura, Yoshiyuki Ozono, Yusuke Ishida, Yasumitsu Takimoto, Manabu Taniguchi, Kazuya Ohata, Yoshihisa Koyama, Takao Imai, Tetsuo Morihana, Makoto Kondo, Takashi Sato, Hidenori Inohara, Shoichi Shimada

**Affiliations:** 1 Department of Neuroscience and Cell Biology, Graduate School of Medicine, Osaka University, 2-2 Yamadaoka, Suita, Osaka 565-0871 Japan; 2 Department of Otorhinolaryngology-Head and Neck Surgery, Graduate School of Medicine, Osaka University, 2-2 Yamadaoka, Suita, Osaka 565-0871 Japan; 3 Division of Anatomy and Cell Biology, Tohoku Medical and Pharmaceutical University, 4-4-1, Komatsushima, Aobaku, Sendai, Miyagi 981-8558 Japan; 4 Department of Otorhinolaryngology, Osaka Police Hospital, 10-31, Kitayamacho, Tennoujiku, Osaka 543-0035 Japan; 5 Department of Anatomy and Neuroscience, Graduate School of Medicine, Osaka University, 2-2 Yamadaoka, Suita, Osaka 565-0871 Japan; 6 Department of Otorhinolaryngology, National Hospital Organization Osaka National Hospital, 2-1-14, Hoenzaka, Chuo, Osaka, Osaka 540-0006 Japan

## Abstract

We investigated fibroblast growth factor 12 (FGF12) as a transcript enriched in the inner ear by searching published cDNA library databases. FGF12 is a fibroblast growth factor homologous factor, a subset of the FGF superfamily. To date, its localisation and function in the inner ear have not been determined. Here, we show that FGF12 mRNA is localised in spiral ganglion neurons (SGNs) and the vestibular ganglion. We also show that FGF12 protein is localised in SGNs, the vestibular ganglion, and nerve fibres extending beneath hair cells. Moreover, we investigated FGF12 function in auditory and vestibular systems using *Fgf12*-knockout (FGF12-KO) mice generated with CRISPR/Cas9 technology. Our results show that the inner ear morphology of FGF12-KO mice is not significantly different compared with wild-type mice. However, FGF12-KO mice exhibited an increased hearing threshold, as measured by the auditory brainstem response, as well as deficits in rotarod and balance beam performance tests. These results suggest that FGF12 is necessary for normal auditory and equilibrium function.

## Introduction

Hearing loss is a common problem in people of all ages. The World Health Organization reports that 360 million people worldwide have hearing loss, with 32 million being children^1^. Hearing loss is a major sensory defect, as prelingual hearing loss affects oral language acquisition. As over 50% of prelingual hearing loss is genetic^2^, it is therefore necessary to identify and determine the function of causative genes for hearing loss. Recent studies of transcripts enriched in the inner ear have provided novel insight into hereditary hearing loss and normal auditory function^3,4^. In this study, we focused on fibroblast growth factor 12 (FGF12) as an inner ear-enriched transcript and performed experiments to investigate its localisation and function.

FGF12 is a member of the FGF superfamily, which has 23 FGF members identified to date. FGF11–14 are intracellular non-secreted proteins known as intracellular FGFs or fibroblast growth factor homologous factors (FHFs)^5^. Whereas other FGFs work by binding to FGF receptors (FGFRs), FHFs do not bind to FGFRs^6^. Although reports are limited and much is unknown about FHF function, recent studies have revealed that FHFs bind to and regulate voltage-gated sodium channels (Nav channels)^7,8^. For example, Wittmack *et al*., reported colocalisation between FGF13 and Nav1.6 at nodes of Ranvier in dorsal root axons. Moreover, cell lines in which both proteins were coexpressed showed increases in peak current amplitude^9^. Further, Goldfarb *et al*. overexpressed FGF12 and reported colocalisation with Nav channels in cultured neurons^10^. They also reported that FGF12 single-knockout (KO) mice showed no phenotype, while FGF12/FGF14 double-KO mice exhibited severe ataxia and decreased excitability of cerebellar granule neurons, thereby concluding that FGF12 played an important role together with FGF14 in neuronal action potentials. However, there are no reports showing abnormalities in the auditory or equilibrium function of FGF12-KO mice, and FGF12 function in the inner ear remains to be determined.

In this study, we investigated FGF12 expression and localisation in murine inner ear tissue. Additionally, auditory and vestibular function was examined in the FGF12-KO mice that we generated. Consequently, this is the first report to show FGF12 function in the inner ear, and our findings provide novel insight into the contribution of FHF to normal auditory and vestibular function.

## Results

### FGF12 mRNA is abundantly expressed in the inner ear

**Figure 1:**
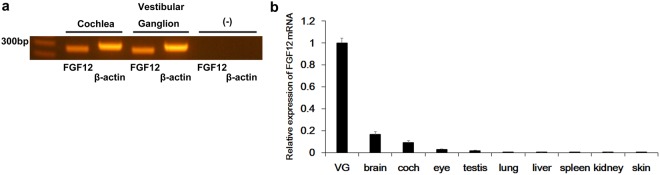
Expression of FGF12 mRNA in the inner ear. (**a**) Detection of FGF12 mRNA expression by reverse transcription-polymerase chain reaction (RT-PCR) in the mouse cochlea and vestibular ganglion. β-Actin positive controls are shown. (−) indicates reaction products without cDNA. These gel images are cropped, and full-length images are presented in Supplementary Information 1. (**b**) Quantitative real-time PCR for FGF12 mRNA using cDNA from various tissues of adult mouse (*n* = 3). Expression levels were normalised to endogenous β-actin. Each expression level was standardised to the vestibular ganglion (VG) and given a value of 1.

We hypothesised that transcripts enriched in the inner ear play a key role in normal hearing and vestibular function. Consequently, investigation of these transcripts will aid understanding of the causes of hereditary hearing loss. cDNA libraries encompassing different tissues contain expression sequence tag (EST) information that enables mRNA expression levels to be compared. Accordingly, we compared GenBank EST clones from inner ear cDNA libraries to those of other tissues. We identified 13 candidate genes specifically expressed in the inner ear. Next, we performed reverse transcription-polymerase chain reaction (RT-PCR) on murine inner ear to confirm that these 13 candidate genes are specifically and abundantly expressed in the inner ear. Among these genes, we found that FGF12 mRNA was abundantly expressed in the cochlea and vestibular ganglion (Fig. 1a). β-Actin was used as an internal control.

To examine the specificity of FGF12 mRNA expression in the inner ear compared with other tissues, we performed quantitative real-time PCR (qPCR) (Fig. 1b). FGF12 mRNA levels were compared in each tissue relative to β-actin as an internal control. FGF12 mRNA levels were highest in the vestibular ganglion, and relatively high in the brain and cochlea. Weak expression was also detected in the eye and testis, with virtually no expression detected in the other tissues examined.

### FGF12 mRNA is localised in spiral and vestibular ganglia

**Figure 2:**
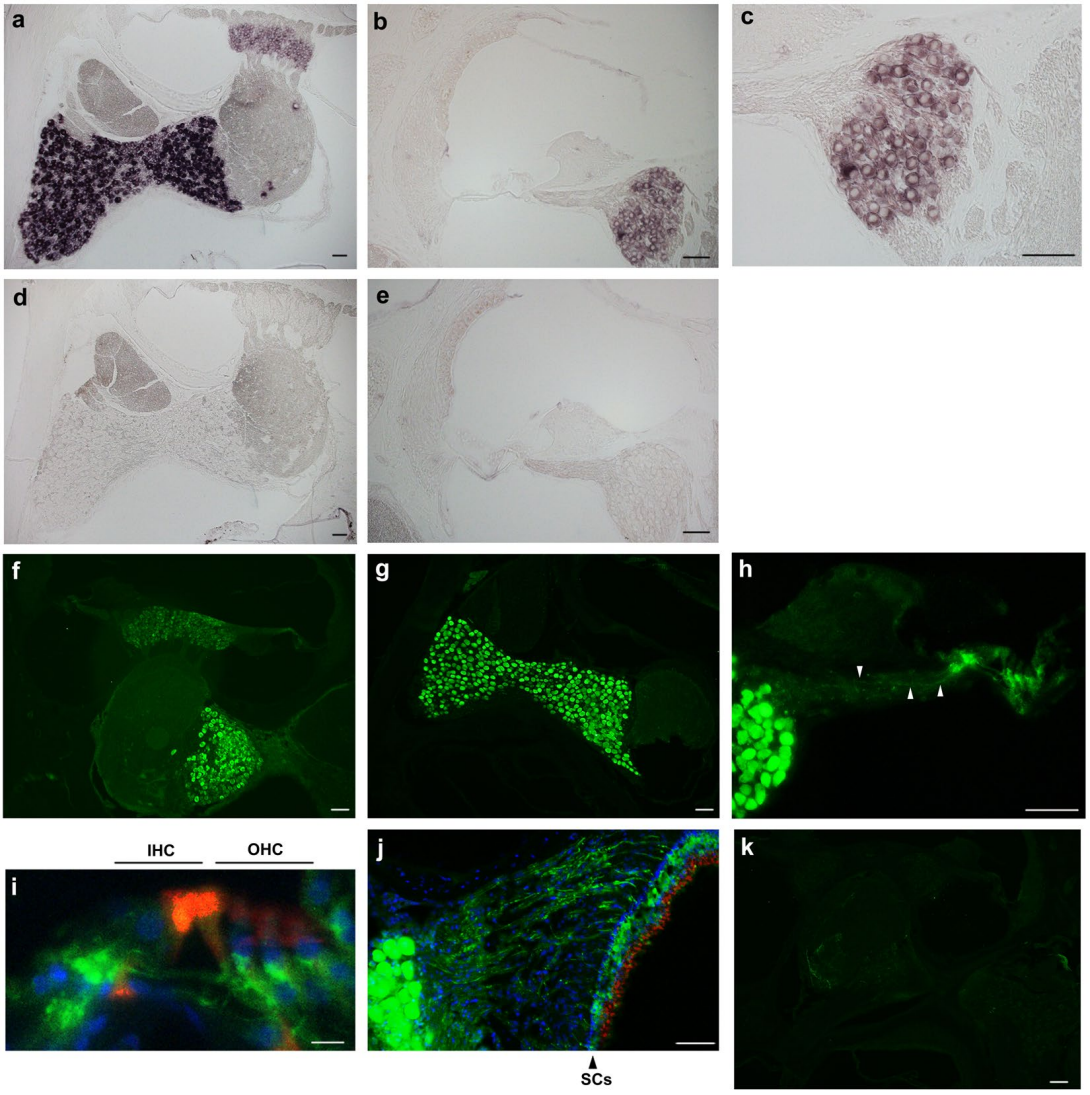
Localisation of FGF12 mRNA and protein expression in the inner ear. (**a**–**c**) FGF12 mRNA localisation in the inner ear by *in situ* hybridisation. (**b**) The cochlea. (**c**) High-magnification image of (**b**), focused on the spiral ganglion. (**d**,**e**) *In situ* hybridisation images using the sense probe as a negative control. (**f**–**j**) Immunofluorescent staining for FGF12 (green) in the inner ear. (**f**) Low-magnification image of the inner ear. (**g**) Superior and inferior regions of the vestibular ganglion. (**h**) The spiral ganglion (SGN) and organ of Corti. White triangles indicate neural fibres. (**i**) Magnified and merged image of the organ of Corti. IHC, inner hair cell; OHC, outer hair cell. (**j**) The vestibular ganglion and sensory epithelium of the saccule. The triangle indicates the layer of supporting cells (SCs), which are characterised by cell alignment and rich cell density. (**k**) Image using FGF12-KO mouse tissue as a negative control. Phalloidin (red) visualises F-actin in hair cells. Hoechst® (blue) visualises the nucleus of each cell. H in (**a**–**g**) and (**i**–**k**), and 10 µm in (**i**).

To examine FGF12 mRNA localisation, we performed *in situ* hybridisation in the inner ear using an FGF12-specific complementary RNA (cRNA) probe. FGF12 mRNA expression was abundantly detected in the spiral and vestibular ganglia (Fig. 2a–c). Further, FGF12 mRNA was expressed in both the superior and inferior regions of the vestibular ganglion. Staining intensity in the vestibular ganglion was stronger compared with the spiral ganglion, confirming our qPCR finding showing that FGF12 mRNA expression is more abundant in the vestibular ganglion (Fig. 1b). No FGF12 mRNA signal was detected in other parts of the inner ear, i.e., hair cells, supporting cells, stria vascularis, or spiral ligaments (Fig. 2b). We also performed *in situ* hybridisation using the sense probe as a negative control, with no staining detected (Fig. 2d,e).

### FGF12 protein is localised in the spiral ganglion, vestibular ganglion, and peripheral nerve fibres of the cochlea and vestibular organ

To examine FGF12 protein localisation, we performed immunohistochemistry in inner ear tissue using an anti-FGF12 antibody. FGF12 protein was expressed in the spiral ganglion and superior and inferior regions of the vestibular ganglion (Fig. 2f,g). This expression pattern corresponded with FGF12 mRNA (Fig. 2a). In addition, higher magnification images of the cochlea showed that FGF12 protein was expressed in nerve fibres (Fig. 2h, white triangles) extending from the spiral ganglion to immediately beneath the inner and outer hair cells (Fig. 2h,i). FGF12 protein was also expressed in nerve fibres of the saccule, extending from the vestibular ganglion through the supporting cell layer to below the hair cells (Fig. 2j). To confirm the specificity and immunoreactivity of the utilized anti-FGF12 antibody, we performed immunohistochemistry on inner ear tissue from FGF12-KO mice. Using the same exposure times, no fluorescent signal was detected (Fig. 2k [cf. Fig. 2f]).

### Generation of FGF12-KO mice using the CRISPR/Cas9 system

**Figure 3:**
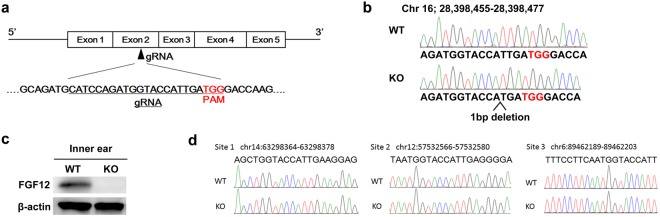
Generation of FGF12-knockout mice by CRISPR/Cas9 technology. (**a**) Diagram of the FGF12 exon 2 sgRNA. The sgRNA sequence is underlined in black. Protospacer adjacent motif (PAM) sequence is indicated in red. (**b**) FGF12 genomic sequence from F3 generation FGF12-knockout (KO) and wild-type (WT) mice. PAM sequence is indicated in red with one base deleted in FGF12-KO mice. (**c**) Western blot analysis of the inner ear from WT and FGF12-KO mice. β-Actin controls are shown below. These blot images are cropped, and full-length images are presented in Supplementary Information 2. (**d**) The genomic sequence around three candidate off-target cleavage sites.

To examine the contribution of FGF12 function to auditory and vestibular function, we generated FGF12-KO mice using the C57BL/6 J strain and clustered regularly interspersed short palindromic repeats (CRISPR)/Cas9 technology. We generated KO mice with a one base-pair (bp) deletion in FGF12 exon 2 (Fig. 3a,b). To confirm FGF12-KO, we performed western blotting using inner ear tissue. In the inner ear of wild-type (WT) mice, a FGF12 band was detected at 20 kDa, which was not present in KO mouse inner ear (Fig. 3c). β-Actin bands were detected as an endogenous control. This result indicated that the single base deletion produced by the CRISPR/Cas9 system could lead to frame shift in FGF12 gene, resulting in deletion of FGF12 protein. In rare cases during genome editing by CRISPR/Cas9 system, the gRNA can recognize another genome sequence similar to the target sequence, thus creating the potential for unexpected genome mutations to occur, known as off-target mutations^11^. To eliminate the possibility of off-target mutations in FGF12-KO mice, we selected three candidate genomic sites with a similar sequence to the small guide RNA (sgRNA). PCR amplification and sequencing of these sites confirmed there were no off-target mutations present in FGF12-KO mice (Fig. 3d).

### Normal cochlear morphology in FGF12-KO mice

FGF12-KO mice seemed to have normal appearance, but exhibited decreased body weight compared with WT mice at 7 weeks of age (KO, 19.69 ± 0.53 g, n = 14; WT, 21.76 ± 0.24 g, n = 17, p < 0.001). Furthermore, FGF12-KO mice seemed to have a normal lifespan, because almost all lived to over 18 months.

**Figure 4:**
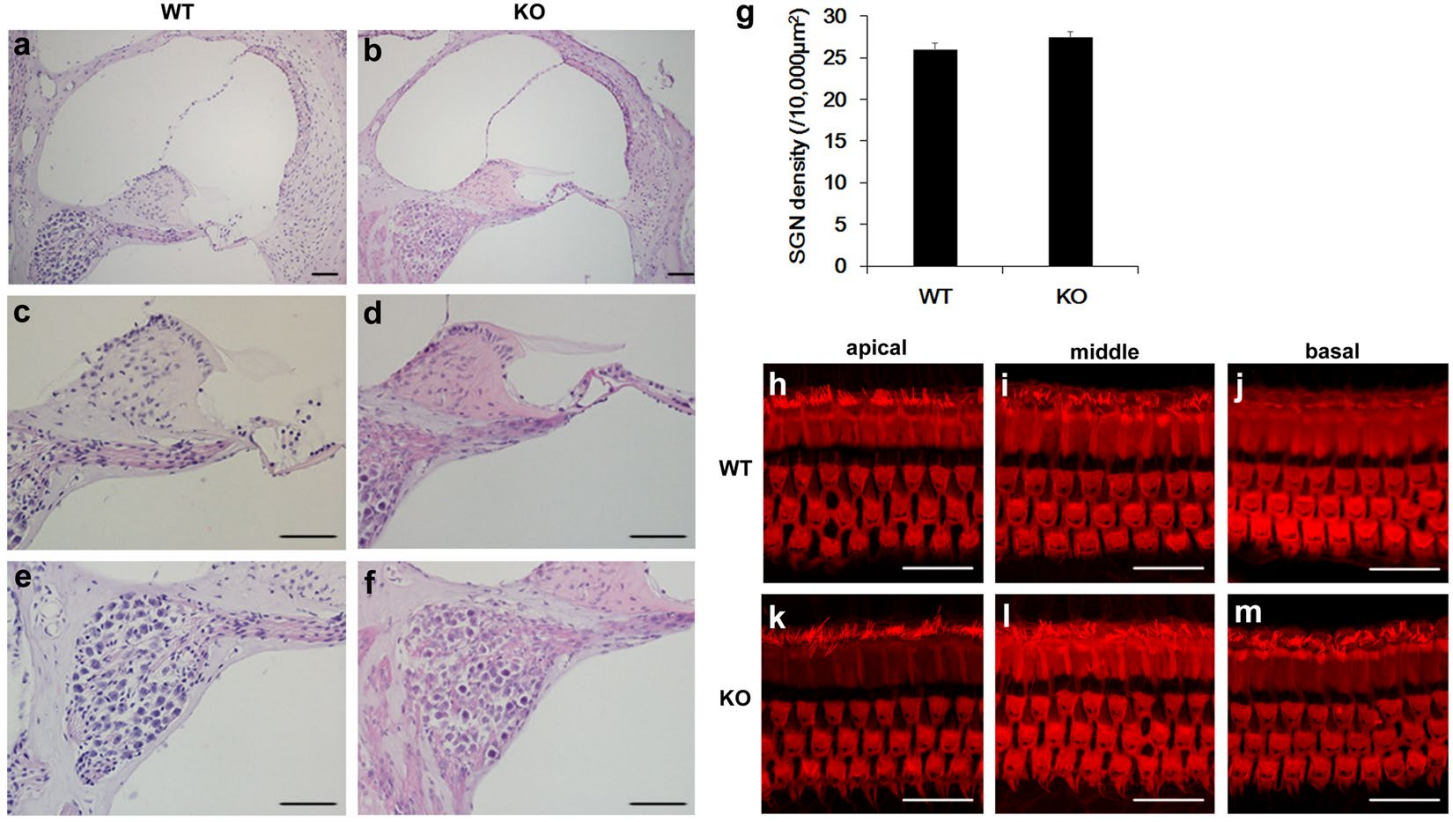
Morphology of FGF12-knockout cochlea. (**a**–**f**) Cochlea sections stained with hematoxylin and eosin from wild-type (WT) (**a**,**c**,**e**) and FGF12-knockout (KO) (**b**,**d**,**f**) mice. (**c**) and (**d**) are high-magnification images of the organ of Corti shown in (**a**) and (**b**), respectively. (**e**) and (**f**) are high-magnification images of spiral ganglion neurons (SGNs) shown in (**a**) and (**b**), respectively. (**g**) SGN density in Rosenthal’s canal of WT (*n* = 4) and FGF12-KO (*n* = 4) cochleae. (**h**–**m**) Confocal images of inner and outer hair cells from WT (**h**–**j**) and FGF12-KO (**k**–**m**) cochlea. Hair cells are stained with rhodamine phalloidin in apical (**h**,**k**), middle (**i**,**l**), and basal (**j**,**m**) turns. Scale bar: 20 µm.

To examine the impact of FGF12 deletion on cochlear development, we compared cochlear morphology in FGF12-KO mice with WT mice. First, we examined cochlear midmodiolar sections stained with haematoxylin and eosin, but detected no obvious differences in spiral ganglion morphology (Fig. 4a–f). Next, we compared the density of spiral ganglion cells within Rosenthal’s canal area in FGF12-KO and WT mice, but again found no significant difference (Fig. 4g). We also examined the morphology of outer and inner hair cells in whole mounts prepared from cochlear tissue of each mouse genotype. Hair cell morphology in FGF12-KO and WT mice was similar from the apical turn to the basal turn of the cochlea (Fig. 4h–m).

### FGF12-KO mice exhibit increased auditory brainstem response thresholds and deficits in rotarod and balance beam performance tests

**Figure 5:**
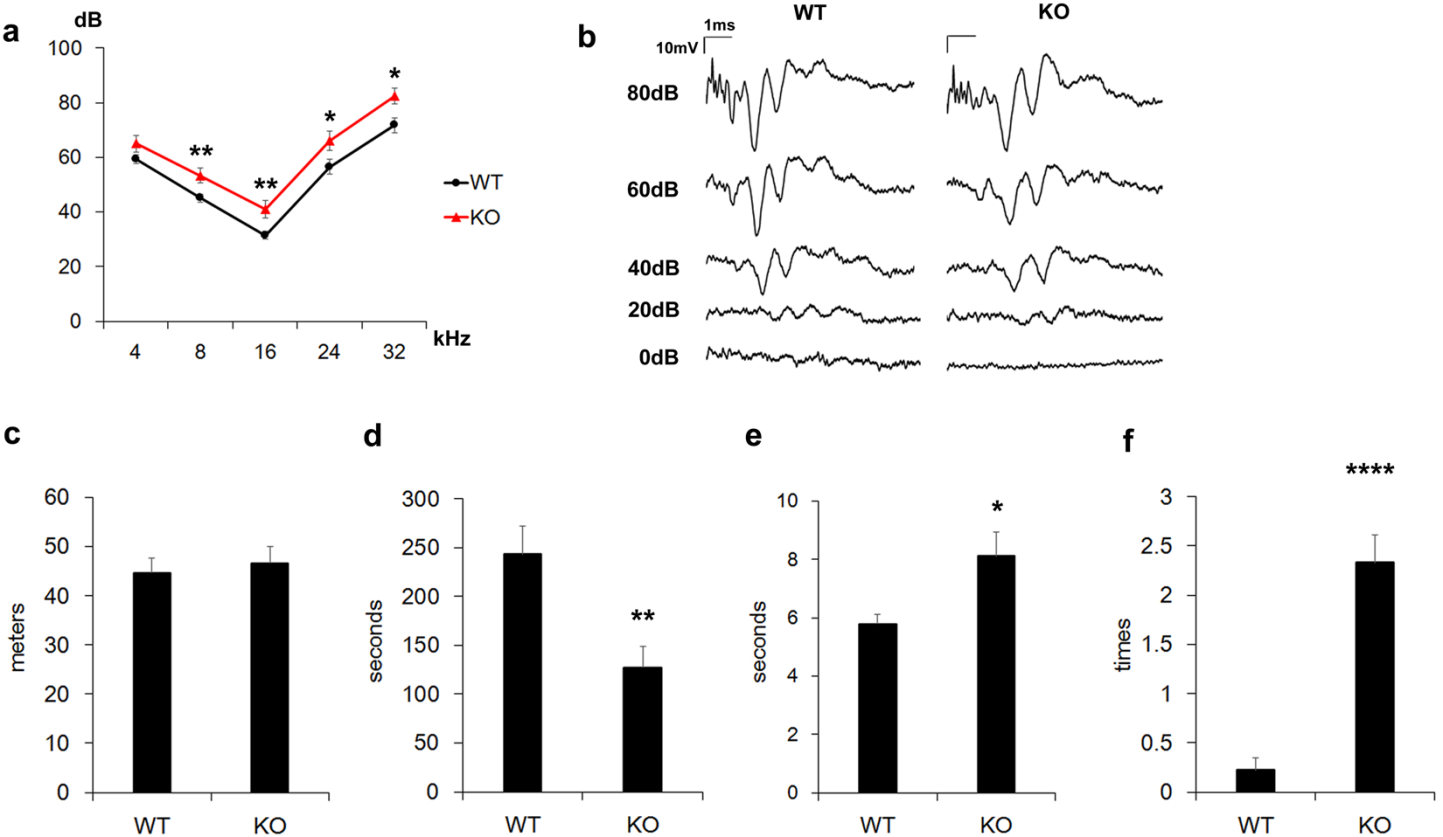
Auditory and behavioural function of FGF12-knockout mice. (**a**) Auditory brainstem response (ABR) in wild-type (WT) (*n* = 17) and FGF12-knockout (KO) (*n* = 14) mice at 7 weeks-of-age. (**b**) ABR waveforms observed with various volumes of click stimuli from WT and FGF12-KO mice. (**c**) Distance travelled in the open field test by WT (*n* = 16) and FGF12-KO (*n* = 13) mice. (**d**) Length of time without falling during the rotarod test in WT (*n* = 16) and FGF12-KO (*n* = 13) mice. (**e**) Time spent to reach the goal in the balance beam test of WT (*n* = 9) and FGF12-KO (*n* = 9) mice. (**f**) Number of slips in the balance beam test of WT (*n* = 9) and FGF12-KO (*n* = 9) mice. *p < 0.05, **p < 0.01, ****p < 0.001 Student’s two-tailed *t*-test.

To examine auditory function, we compared auditory brainstem responses (ABR) in WT and FGF12-KO mice. FGF12-KO mice exhibited increased thresholds in the range of 8 to 32 kHz (4 kHz; *p* = 0.1; 8 kHz; *p* = 0.08; 16 kHz, *p* = 0.008; 24 kHz, *p* = 0.04; 32 kHz, *p* = 0.01) (Fig. 5a). As FGF12 mRNA and protein are expressed in the spiral ganglion, we compared evoked waveforms using click stimuli. However, there was no marked difference in waveforms obtained from WT and FGF12-KO mice (Fig. 5b).

Moreover, we hypothesised that FGF12-KO mice may have a defect in vestibular function because strong FGF12 expression was detected in the vestibular organ. To investigate this, we performed the open field test, rotarod test, and balance beam test on WT and FGF12-KO mice. During the open field test, FGF12-KO mice exhibited normal movement. Further, the entire distance covered was similar to WT mice (*p* = 0.07) (Fig. 5c). However, during the rotarod test, FGF12-KO mice fell off the wheel significantly earlier than WT mice (*p* = 0.005) (Fig. 5d). While in the balance beam test, FGF12-KO mice took longer to reach the goal than WT mice (*p* = 0.02) (Fig. 5e) and tended to slip from the bar at a significantly higher frequency compared with WT mice (*p* < 0.001) (Fig. 5f).

### mRNA expression and localisation of other FHFs in the inner ear

**Figure 6:**
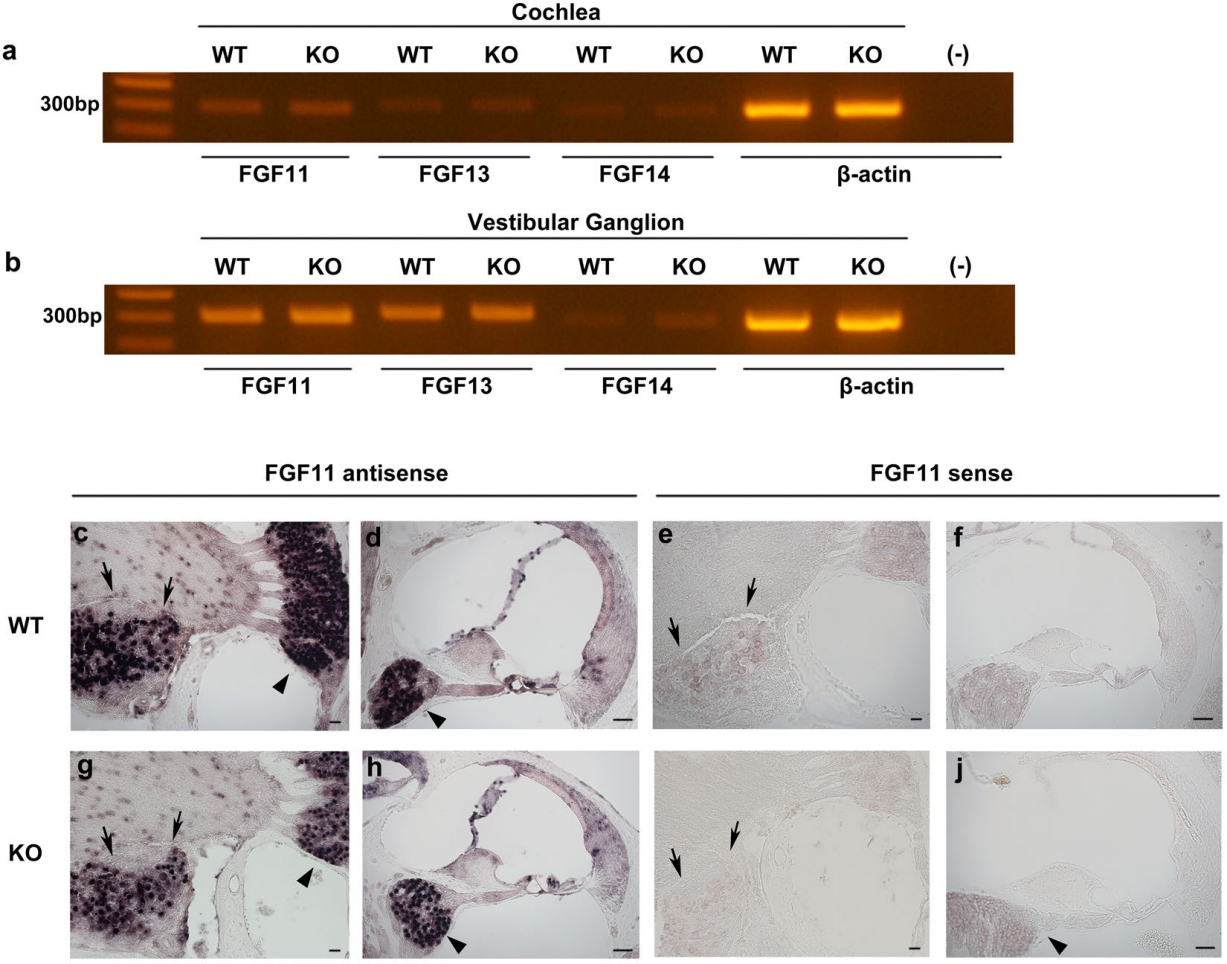
Expression and localisation of other FHFs mRNA in the inner ear. (**a**) Detection of FGF11, FGF13, and FGF14 mRNA expression by RT-PCR in cochleae of WT and FGF12-KO mice. β-actin positive controls are shown. (−) indicates reaction products without cDNA as a negative control. These gel images are cropped, and full-length images are presented in Supplementary Information 3. (**b**) Detection of FGF11, FGF13, and FGF14 mRNA expression by RT-PCR in vestibular ganglia of WT and FGF12-KO mice. (**c**,**d**) FGF11 mRNA localisation in the WT mouse inner ear by *in situ* hybridisation. (**e**,**f**) *In situ* hybridisation images on the WT mouse inner ear using the sense probe as a negative control. (**g**,**h**) FGF11 mRNA localisation in the FGF12-KO mouse inner ear by *in situ* hybridisation. (**i**,**j**) *In situ* hybridisation images of the FGF12-KO mouse inner ear using the sense probe as a negative control. Black triangles indicate spiral ganglions, and black arrows indicate vestibular ganglions in (**c**–**j**). Scale bar: 50 µm.

FHFs (FGF11–14) share common domains^12^ and might compensate for each other. Thus, we performed RT-PCR and *in situ* hybridisation of other FHFs (FGF11, FGF13, and FGF14) in WT mice. RT-PCR (Fig. 6a,b) revealed FGF11 mRNA expression in the cochlea and vestibular ganglion, while FGF13 mRNA was expressed in the vestibular ganglion, but only faintly in the cochlea. FGF14 mRNA expression was only faintly observed in both the cochlea and vestibular ganglion. Among other FHFs, FGF11 mRNA was expressed in the spiral and vestibular ganglia similar to FGF12 expression. In addition, FGF11 was also expressed in sensory hair cells and Reissner’s membrane (Fig. 6c–f). We also performed RT-PCR (Fig. 6a,b) and *in situ* hybridisation (Fig. 6g–j) of FGF11 on FGF12-KO mouse tissue. We could not detect a difference compared with WT mice (Fig. 6).

## Discussion

Here, we focused on FGF12 as an enriched inner ear transcript. We found that FGF12 mRNA is abundantly expressed in the inner ear (Fig. 1) and localised in the vestibular and spiral ganglia (Fig. 2). Further, we show that FGF12 protein is expressed in both ganglia and peripheral nerve fibres in the cochlea and vestibular organ (Fig. 2). We also investigated FGF12 function using FGF12-KO mice (Fig. 3) and found increased ABR thresholds and behavioural test deficits in these animals (Fig. 5). Altogether, our data suggest that FGF12 plays a key role in auditory and vestibular organs.

In this study, we generated FGF12-KO mice using CRISPR/Cas9 technology^11^. FGF12 protein consists of 181 amino acids and its open reading frame contains 546 bases. FGF12 mRNA contains 5 exons, with the functional domain spanning from exon 2 to exon 4, thus including the 13^th^ amino acid (threonine) to the 136^th^ amino acid (phenylalanine)^13^. FGF12-KO mice used in this study have a single base deletion of the 92^nd^ base (thymine) in the open reading frame of 546 bases. This point mutation results in a frameshift in protein translation, as well as the appearance of a stop codon following the 45^th^ amino acid of the entire FGF12 protein; thus, protein translation stops at this point. As a result, the mutated protein contains only a small part of the functional domain, which comprises the first 18 of 124 amino acids. Moreover, it disrupts the three-dimensional structure of FGF12 protein, which consists of twelve β-trefoils located from exon 2 to exon 4^6^, as the mutated protein has only two of the twelve β-trefoils of native FGF12 protein. Thus, we propose that with only two β-trefoils, FGF12 could not form its functional three-dimensional structure. Notably, although we could not completely deny the existence of premature FGF12 protein, which contains only exons 1 and 2, the premature protein lacks almost all of the functional domain, suggesting that the premature protein does not have FGF12 function.

Mammalian SGNs transmit sound stimuli from hair cells to the brain. SGN cell bodies lie within Rosenthal’s canal in the cochlea, with their peripheral processes extending to the organ of Corti to form synapses with hair cells. Alternatively, SGN central processes project to the auditory nerve^14^. SGNs are the first neurons of auditory neural pathways and many studies have shown that proteins expressed in SGNs are responsible for hearing loss^15,16^. For example, Salehi *et al*. reported that neuropilin-1 (Nrp1) is localised in SGN cells, with the cochlea of *Nrp1* conditional-KO mice showing significantly lower SGN density and aberrant axons. *Nrp1* conditional-KO mice also exhibited increased ABR thresholds compared with WT mice. Similarly, in this study, FGF12-KO mice showed increased ABR thresholds, but almost the same SGN density and cochlear morphology as WT mice.

In a recent study, FGF12 single-KO mice showed no phenotype, while FGF12/FGF14 double-KO mice developed severe ataxia and decreased neuronal excitability. In our study, FGF12-KO mice showed a shift in ABR threshold within 15 dB (Fig. 5a), as long as FGF12 was abundantly expressed in the spiral ganglion and neural fibres of the organ of Corti. To examine the ability of other FHFs to compensate for FGF12, we examined mRNA expression of other FHFs (FGF11, FGF13, and FGF14) using RT-PCR (Fig. 6a,b), as well as localisation by *in situ* hybridisation (Fig. 6c–j) in WT and FGF12-KO mice tissue. Our results indicated that FGF11 mRNA was localized in the spiral and vestibular ganglia where FGF12 mRNA was localized (Fig. 6c–j); thus, it might be possible that FGF11 could compensate for reduced or lost FGF12 function in the inner ear. Further investigation of FGF11 function in the inner ear is needed, for example, experiments using FGF12 and FGF11 double-KO mice.

In terms of auditory function, each part of the ABR waveform represents neuronal excitement of a specific part of the auditory pathway^17^. Peak I is generated in the spiral nerve, peak II in the cochlear nucleus, and peaks III–IV in the more central pathway. Generally, if the auditory central pathway is significantly impaired, waveforms of peaks II–V would be different. In this study, peak II–V waveforms of FGF12-KO mice and WT mice looked similar (Fig. 5b). Therefore, we predicted that loss of FGF12 does not dramatically affect the auditory central pathway. However, the possibility for participation of the central pathway is not completely excluded. Further investigation of auditory function in the central nervous system of FGF12-KO mice is needed. We also performed rotarod and balance beam performance tests using FGF12-KO mice, and found deficits in both behavioural tests. FGF12 is abundantly expressed in the vestibular ganglion and neural fibres beneath hair cells. Therefore, deficits may be present in the peripheral vestibular system of FGF12-KO mice. Nonetheless, deficits in the rotarod and balance beam performance tests may occur when mice have insufficient visual or cerebellar function; thus, other experimental systems and electrophysiological analysis are necessary to determine the exact pathology. Moreover, further investigation is needed to determine vestibular function in FGF12-KO mice. Such results could provide novel insight into diseases involving dizziness, especially congenital dizziness.

In this study, we showed that FGF12 mRNA and protein are expressed in auditory and vestibular peripheral neurons, and that FGF12-KO mice exhibit elevated ABR thresholds and deficits in the rotarod and balance beam performance tests. However, further studies are needed to determine the underlying mechanisms that cause these changes. Nonetheless, our results will aid understanding of hereditary hearing loss and dizziness diseases.

## Methods

The Institute of Experimental Animal Sciences of Faculty of Medicine at Osaka University approved all animal procedures in this study. The protocol for these experiments, including genomic recombination, was reviewed and approved by The Institute of Experimental Animal Sciences of Faculty of Medicine at Osaka University (No. 27-010-019).

### Animals

All animal experiments were performed in accordance with institutional guidelines set by the Osaka University School of Medicine Animal Care and Use Committee. C57BL/6 J mice were purchased from Japan SLC (Hamamatsu, Japan). Male mice were used for experiments at 7 weeks of age.

### RT-PCR

Total RNA was isolated from whole cochlea, vestibular ganglia, and the brain temporal lobe using the RNA extraction reagent NucleoSpin® (Macherey-Nagel, Duren, Germany). cDNA synthesis was performed using a QuantiTect Reverse Transcription Kit (Qiagen, Valencia, CA, USA). PCR was performed on a S1000 thermal cycler (Bio-Rad, Hercules, CA, USA), using the following primers: FGF12 (254-bp product length), 5′-CTACACCCTCTTCAATCTAATTCC-3′ (sense) and 5′-TTCCCCTTCATGATTTGACC-3′ (antisense); β-actin (298-bp product length), 5′-GATCCTGACCGAGCGTGGCTACA-3′ (sense) and 5′-CGGATGTCAACGTCACACTTCA-3′ (antisense); FGF11 (313-bp product length), 5′-TAGCCTGATCCGACAGAAGC-3′ (sense) and 5′-TGAAGTGGGTGAAGGAGCTG-3′ (antisense); FGF13 (315-bp product length), 5′-CGAGGACAGCACTTACACTCTG-3′ (sense) and 5′-AGTGGTTTGGGCAGAAAATG-3′ (antisense); and FGF14 (288-bp product length), 5′-CCAATTCCACACTGTTCAACC-3′ (sense) and 5′-GAGCTGCTGGTTTGGTTTTC-3′ (antisense). PCR parameters were: 2 min at 95 °C, 30 cycles of 45 sec at 95 °C, 45 sec at 56 °C, and 18 sec at 72 °C, followed by 5 min at 72 °C.

### qPCR

Total RNA was isolated from the brain and several internal organs of adult mice using NucleoSpin. qPCR was performed using a QuantiTect SYBR Green PCR Kit (Qiagen). qPCR conditions were: 95 °C for 15 min followed by 40 cycles of 15 s at 95 °C, 30 s at 56 °C, and 30 s at 72 °C. The same FGF12 and β-actin primers were used as for RT-PCR. Relative gene expression was determined using β-actin expression as an endogenous internal control.

### Cochlea dissection

The entire inner ear tissue (including the cochlea and vestibular apparatus) was dissected from the skull of adult mice. Cochlea dissection and tissue preparation for cryosections were performed as previously described^18^. Cochleae were decalcified for 7 days. Cochleae used for *in situ* hybridisation and immunostaining with anti-Pan-Nav antibody were decalcified for 3 days to enable RNA probe and antibody reactivity. Frozen cochlea compounds were cut into 10-µm sections using a cryostat and then adhered to slides (PRO-01; Matsunami, Osaka, Japan).

For whole mount cochlea preparations, adult mice cochleae were isolated from the temporal bones and immersed in 4% paraformaldehyde (PFA) in 0.1 M phosphate-buffered saline (PBS) overnight, then decalcified in PBS containing 10% ethylenediaminetetraacetic acid (EDTA) for 7 days. Otic capsules were microscopically removed with forceps and a scalpel in PBS.

To evaluate SGN density in cochleae, 10-µm frozen sections were stained with Harris hematoxylin and eosin. SGN cell counts were obtained from 10 midmodiolar sections of each cochlea by an observer blinded to the experimental group. The size of Rosenthal’s canal area was measured using ImageJ software^19^. SGN density of each cochlea was compared using Student’s two-tailed *t*-test.

### *In situ* hybridisation

To prepare RNA probes, plasmids containing the entire cDNA sequence of FGF12 were used (IMAGE clone 5369273; Source Bioscience, Nottingham, UK). To generate FGF12 probes, 1248 bp of FGF12 cDNA was excised using *EcoR*V and *HindIII*, and the fragment was ligated into the same plasmid vector digested with *EcoRV* and *HindIII*. FGF12 antisense probe with a common sequence was generated using ligated cDNA clone digested with *HindIII* and SP6 RNA polymerase. The sense probe was generated using *EcoRV* and T7 RNA polymerase. To generate probes for FGF11, FGF13, and FGF14, we performed PCR amplification of specific regions of purchased cDNAs, and confirmed that all PCR products were precisely synthesized using Sanger sequencing. We purchased FGF11 cDNA (IMAGE clone 30101881; Source Bioscience), FGF13 cDNA (D130019P14; DNAform, Yokohama, Japan), and FGF14 cDNA (9630023C21; DNAform). Primers used for PCR amplification included: FGF11 (1198-bp product length), 5′-CCTAGGCCTGGACAAGGAAG-3′ (sense) and 5′-TTGTCTCCCTGGCAGAACC-3′ (antisense); FGF13 (779-bp product length), 5′-ACCAAACTATACAGCCGACAAG-3′ (sense) and 5′-TTTGGCAGTCCTTCTTCCAG-3′ (antisense); and FGF14 (750-bp product length), 5′-CCCAGCTCAAGGGCATAG-3′ (sense) and 5′-CTGGTTTGTCCAGGTGTCTTC-3′ (antisense). Synthesized products were ligated into pGEM^®^-T Easy Vector (Promega, Tokyo, Japan). FGF11 antisense probe was generated using ligated cDNA clone digested with *SalI* and T7 RNA polymerase, and sense probe with *NcoI* and SP6 RNA polymerase. FGF13 and FGF14 antisense probes were generated using ligated cDNA clones digested with *NcoI* and SP6 RNA polymerase, and sense probes with *SalI* and T7 RNA polymerase. All probes were labelled using the DIG RNA labelling kit (Roche, Indianapolis, IN, USA), according to the manufacturer’s protocol.

*In situ* hybridisation was performed as described previously^20^. Images were viewed using a BX53 microscope (Olympus, Tokyo, Japan).

### Phalloidin immunolabelling

To label and visualise cochlea hair cells, whole mount preparations of organs of Corti were stained with phalloidin, as previously described^20^. Slides were observed by laser-scanning confocal microscopy (LSM710; Carl Zeiss, Jena, Germany).

### Immunofluorescence staining

Sections were incubated with primary antibodies in blocking solution containing 5% bovine serum albumin and 0.3% Triton X-100 in PBS at 4 °C overnight. Fluorescent-labelled secondary antibodies (1:500) (Alexa Fluor® 488 and 594, Thermo Fisher Scientific, Yokohama, Japan) in PBS were incubated with sections for 1 h at room temperature. For nuclear staining, Hoechst® 33342 (1:1000) (Thermo Fisher Scientific) in PBS was incubated with sections for 2 min. To stain F-actin in cochlear hair cells, Alexa Fluor® 594 Phalloidin (1:500) (Thermo Fisher Scientific) was incubated in PBS for 30 min.

Primary antibodies used included polyclonal rabbit anti-FGF12 (1:500) (13784-1-AP; Proteintech, Rosemont, USA) and polyclonal guinea pig anti-Pan-Nav (1:400) (ASC-003; Alomone Labs, Jerusalem, Israel).

### Generation of FGF12-KO mice

FGF12-KO mice were generated using CRISPR/Cas9 technology with the assistance of NPO Biotechnology Research and Development^11^. An sgRNA targeting FGF12 exon 2 was synthesised *in vitro*. C57BL/6 female mice were superovulated, then oocytes were collected from ampullae and co-incubated with sperm from C57BL/6 male mice. Pronuclear stage eggs were injected using a micromanipulator with pX330 plasmid containing hCas9 and sgRNA (5′-CATCCAGATGGTACCATTGATGG-3′) at 5 ng/µl. Fertilised eggs were transferred into the oviducts of pseudopregnant ICR females. F0 offspring were genotyped by PCR amplification and direct sequencing. Altogether, 12 of 28 offspring carried non-mosaic genomic mutations, while five carried mosaic genomic mutations. One F0 mosaic mutant mouse was bred with a C57BL/J mouse to derive F1 heterozygous non-mosaic mutant mice. Homozygous mutant F2 offspring carrying a 1-bp deletion were bred from F1 heterozygous parents. Next, homozygous mutant F2 offspring were backcrossed to each other. FGF12 homozygous-KO female mice generated few newborn mice, and therefore KO offspring from heterozygous female mice were also used. All neonatal mice were genotyped by PCR and Sanger sequencing using the following primers: 5′-CCGAGTGCTGGAAGTAAAGG-3′ (sense) and 5′-ATTGCAAGTAGCTGGCGTTC-3′ (antisense), which produced a 454-bp product. For off-target analysis, the CRISPR direct website^21^ was used to identify candidates for off-target sites. The most similar sites, with 14 or 15 bases of 23 bases corresponding with the sgRNA sequence, were selected for sequence analysis.

### Western blotting

Whole inner ear and brain temporal lobe of each mouse was homogenised in buffer containing 50 mM Tris-HCl (pH 7.5), 150 mM NaCl, 5 mM EDTA, and 1% Triton X-100. After centrifugation at 17,700 × *g* for 45 min, supernatants were collected. FGF12 was detected with an anti-FGF12 antibody (1:1000) raised against amino acids 1–181 of human FGF12 (13784-1-AP; Proteintech), followed by reaction with a horseradish peroxidase-labelled anti-rabbit IgG secondary antibody (1:3000) (Promega). Pierce™ ECL Plus Western Blotting Substrate (Thermo Fisher Scientific) was used for visualisation. As an endogenous control, the same membrane was probed with anti-β-actin antibody (1:10000) (ab8227; Abcam, Cambridge, UK).

### Auditory brainstem response

Mice were anaesthetised by intraperitoneal injection of ketamine (100 mg/kg) and xylazine (10 mg/kg), and placed into a sound-isolated chamber. Subcutaneous needle electrodes were inserted into the pinna and vertex, with a ground electrode near the hip. Responses to click stimuli and tone pip stimuli at 4, 8, 12, 24, and 32 kHz were recorded using a Power Lab 2/25 (AD Instruments, Sydney, Australia) and TDT Auditory Workstation (Tucker-Davis Technologies, Alachua, FL, USA). The duration of tone bursts was 1 msec. The sound level was raised in 5-dB steps from 0 dB to 100 dB. Overall, 500 responses were amplified and averaged. The threshold level was determined as the point above which any wave could be detected. All ABR were measured blinded to mouse genotype.

### Open field test

The open field test was used to examine locomotor activity and emotionality. The experimental protocol has been previously described^22^. Briefly, mice were placed in the centre of a square open field box (50 × 50 × 40 cm; Muromachi Kikai, Tokyo, Japan) and total distance travelled was recorded using an ANY-mazeTM video tracking system (Stoelting Co., Wood Dale, IL, USA)^23^. Movement of mice was recorded for 10 min.

### Rotarod test

The rotarod test (MK-610A; Muromachi Kikai, Tokyo, Japan) was used to assess balance, grip strength, and motor coordination. The apparatus consisted of a rotating rod that accelerated from 4 to 30 rpm over 5 min, and then continued for a further 5 min. Two trials were tested at 30 min intervals during daytime. After each trial, the apparatus was cleaned and dried. The time that mice remained on the rotating rod without falling off was measured.

### Balance beam test

The balance beam test was used to evaluate motor coordination and balance. This protocol was based on past studies^24,25^. Briefly, a 1-m-long wood beam (20-mm diameter) was placed 50 cm above a desktop, and a small masked box was placed at one of its ends as the finish point. The start point was set at 75 cm from the finish point. First, the mouse was placed in the box for 30 sec. For training, the mouse was allowed to cross the beam three times before each beam test. After training, the time to reach the finish point and number of slips was scored. The test was performed two times at 10-min intervals, and the average score of both trials was determined.

### Data availability

All data obtained or analysed during this study are included in this manuscript. Raw data are available from the corresponding author on any reasonable request.

## Electronic supplementary material

Supplementary Information

## References

[1] World Health Organization Media Centre. Deafness and hearing loss. http://www.who.int/news-room/fact-sheets/detail/deafness-and-hearing-loss (2018)

[2] Marazita ML, Brenda Rawlings LMP, Remington E, Amos KS, Nance WE (1993). Genetic Epidemiological Studies of Early-Onset Deafness in the U.S. School-Age Population. Am. J. Med. Genet..

[3] Asamura K (2005). Type IX collagen is crucial for normal hearing. Neuroscience.

[4] Hildebrand MS (2007). Gene expression profiling analysis of the inner ear. Hear. Res..

[5] Ornitz DM, Itoh N (2015). The Fibroblast Growth Factor signaling pathway. Wiley Interdiscip Rev Dev Biol.

[6] Olsen SK (2003). Fibroblast growth factor (FGF) homologous factors share structural but not functional homology with FGFs. J. Biol. Chem..

[7] Wang C, Wang C, Hoch EG, Pitt GS (2011). Identification of novel interaction sites that determine specificity between fibroblast growth factor homologous factors and voltage-gated sodium channels. J. Biol. Chem..

[8] Liu CJ, Dib-Hajj SD, Renganathan M, Cummins TR, Waxman SG (2003). Modulation of the cardiac sodium channel Nav1.5 by fibroblast growth factor homologous factor 1B. J. Biol. Chem..

[9] Wittmack EK (2004). Fibroblast growth factor homologous factor 2B: association with Nav1.6 and selective colocalization at nodes of Ranvier of dorsal root axons. J. Neurosci..

[10] Goldfarb M (2007). Fibroblast growth factor homologous factors control neuronal excitability through modulation of voltage-gated sodium channels. Neuron.

[11] Mashiko D (2013). Generation of mutant mice by pronuclear injection of circular plasmid expressing Cas9 and single guided RNA. Sci. Rep..

[12] Goldfarb M (2005). Fibroblast growth factor homologous factors: evolution, structure, and function. Cytokine Growth Factor Rev..

[13] European Bioinformatics Institute. Pfam. https://pfam.xfam.org/. (2017).

[14] Nayagam BA, Muniak MA, Ryugo DK (2011). The spiral ganglion: connecting the peripheral and central auditory systems. Hear. Res..

[15] Sato T (2006). Progressive hearing loss in mice carrying a mutation in the p75 gene. Brain Res..

[16] Salehi P (2017). Role of Neuropilin-1/Semaphorin-3A signaling in the functional and morphological integrity of the cochlea. PLoS Genet.

[17] Henry KR (1979). Auditory brainstem volume-conducted responses: origins in the laboratory mouse. J. Am. Aud. Soc..

[18] Whitlon DS, Mary RS, Greinera A (2001). Cryoembedding and sectioning of cochleas for immunocytochemistry and *in situ* hybridization. Brain Res Brain Res Protoc..

[19] Schneider CA, Rasband WS, Eliceiri KW (2012). NIH Image to ImageJ: 25 years of image analysis. Nature Methods.

[20] Hanada Y (2017). Epiphycan is specifically expressed in cochlear supporting cells and is necessary for normal hearing. Biochem. Biophys. Res. Commun..

[21] Naito Y, Hino K, Bono H, Ui-Tei K (2015). CRISPRdirect: software for designing CRISPR/Cas guide RNA with reduced off-target sites. Bioinformatics.

[22] Nakatani J (2009). Abnormal behavior in a chromosome-engineered mouse model for human 15q11-13 duplication seen in autism. Cell.

[23] Kondo M, Nakamura Y, Ishida Y, Shimada S (2015). The 5-HT3 receptor is essential for exercise-induced hippocampal neurogenesis and antidepressant effects. Mol. Psychiatry.

[24] Carter, R. J., Morton, J. & Dunnett, S. B. Motor coordination and balance in rodents. *Curr*. *Protoc*. *Neurosci*. Chapter 8, Unit 8 12, 10.1002/0471142301.ns0812s15 (2001). 10.1002/0471142301.ns0812s15 18428540

[25] Romand R (2013). Retinoic acid deficiency impairs the vestibular function. J. Neurosci..

