# Supplementary material for: Fibroblast growth factor 12 is expressed in spiral and vestibular ganglia and necessary for auditory and equilibrium function
Source: Sci Rep. 2018 Jul 31;8:11491. doi: 10.1038/s41598-018-28618-0 (PMC6068167; doi:10.1038/s41598-018-28618-0)
Supplement: Supplementary file 1 — Supplementary Information [file 41598_2018_28618_MOESM1_ESM.pdf]

SUPPLEMENTARY INFORMATION

Fibroblast growth factor 12 is expressed in spiral and vestibular ganglia and necessary for auditory and equilibrium function

Yukiko Hanada, Yukiko Nakamura, Yoshiyuki Ozono, Yusuke Ishida,
Yasumitsu Takimoto, Manabu Taniguchi, Kazuya Oahata,
Yoshihisa Koyama, Takao Imai, Tetsuo Morihana, Takashi Sato,
Hidenori Inohara, Shoichi Shimada

Supplementary Information 1.

Full-length gel cropped for representative figure.

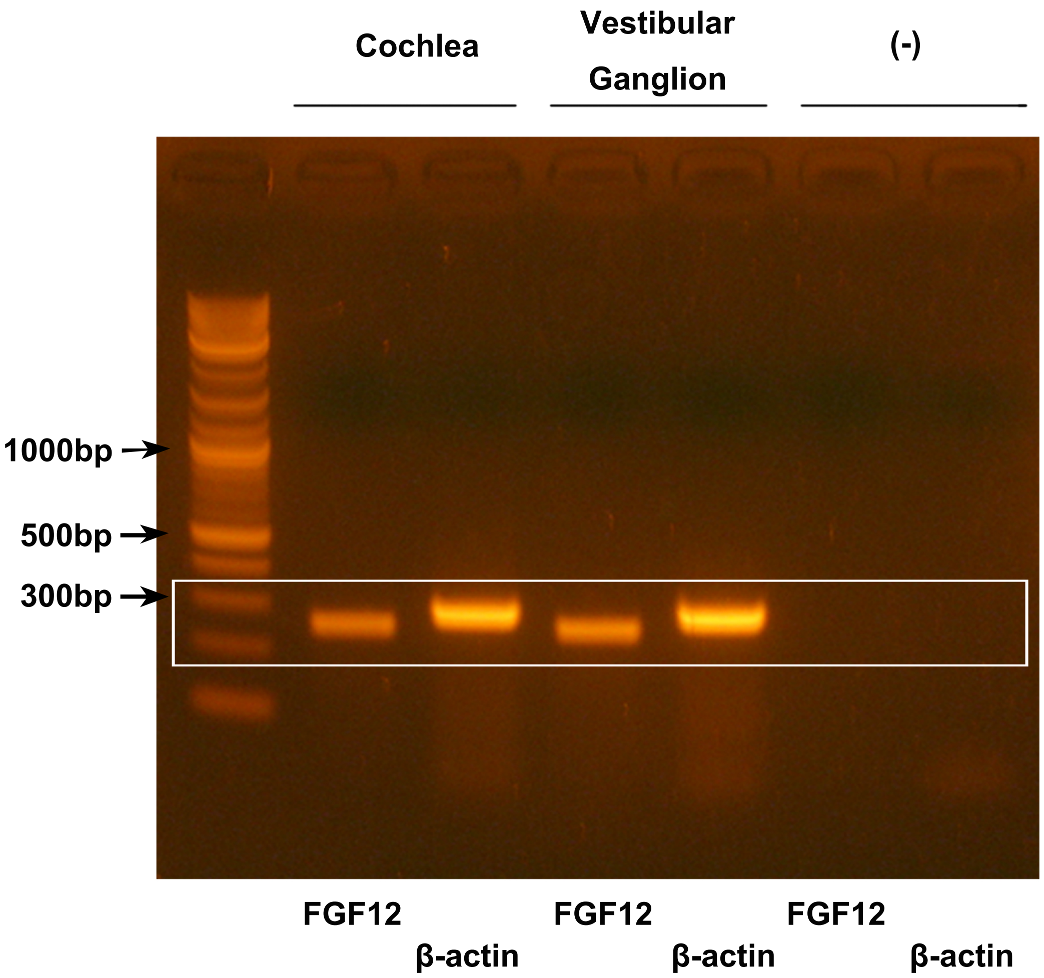

Figure. S-1.

Full-length gel for electrophoresis shown in Figure 1a.

Boxes indicate lanes that were used in Figure 1a.

Supplementary Information 2.

Full-length blots cropped for representative figures.

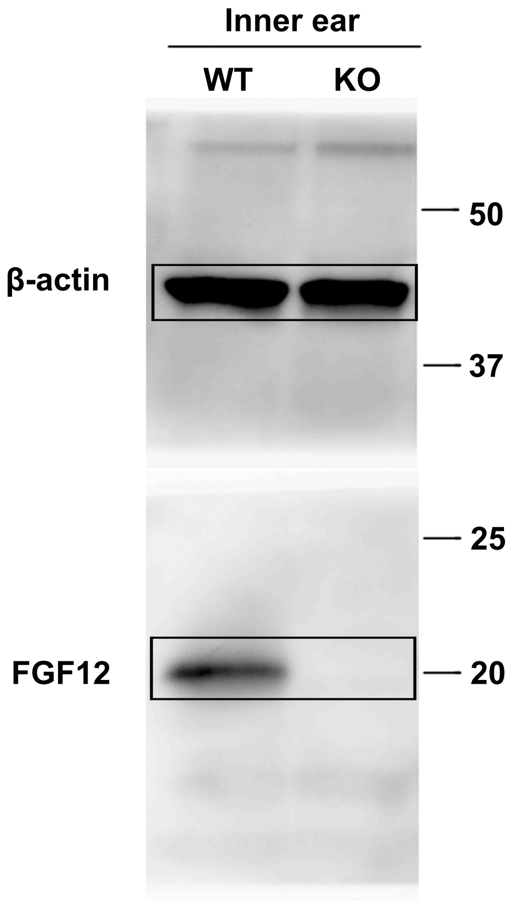

Figure S-2.

Full-length of immunoblots shown in Figure 3c.

Boxes indicate lanes that were used in Figure 3c.

The membrane is cut into two parts for each antibody.

Supplementary Information 3.

Full-length gels cropped for representative figures.

Fig.6a

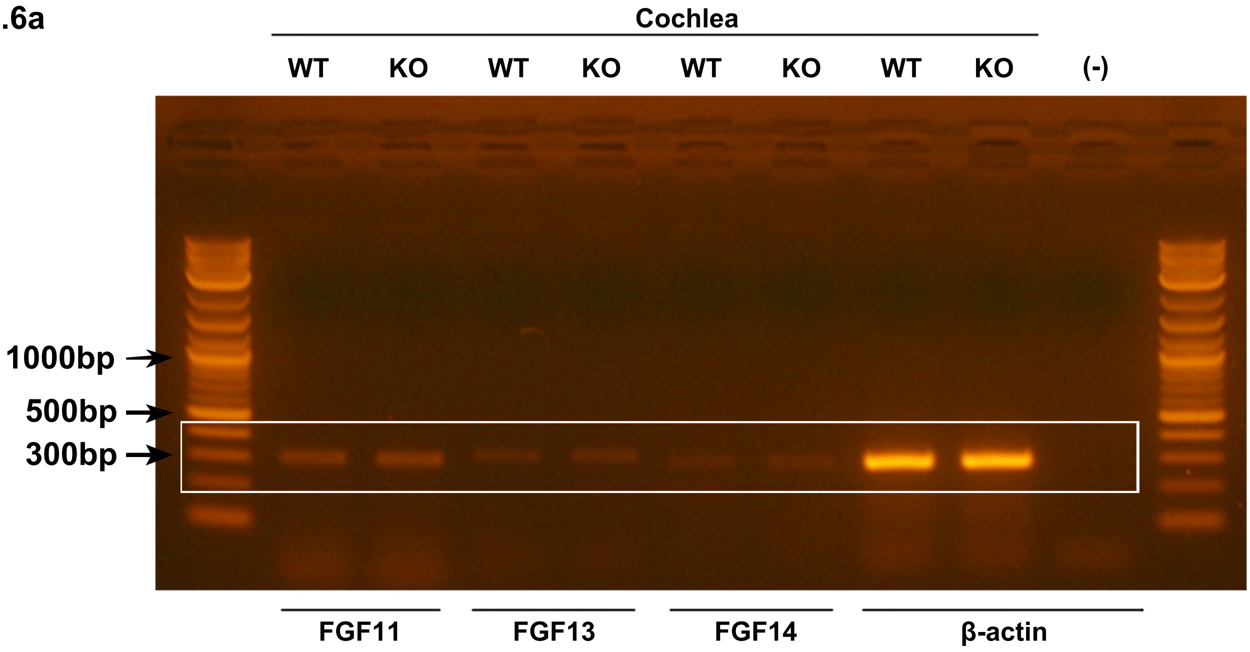

Fig.6b

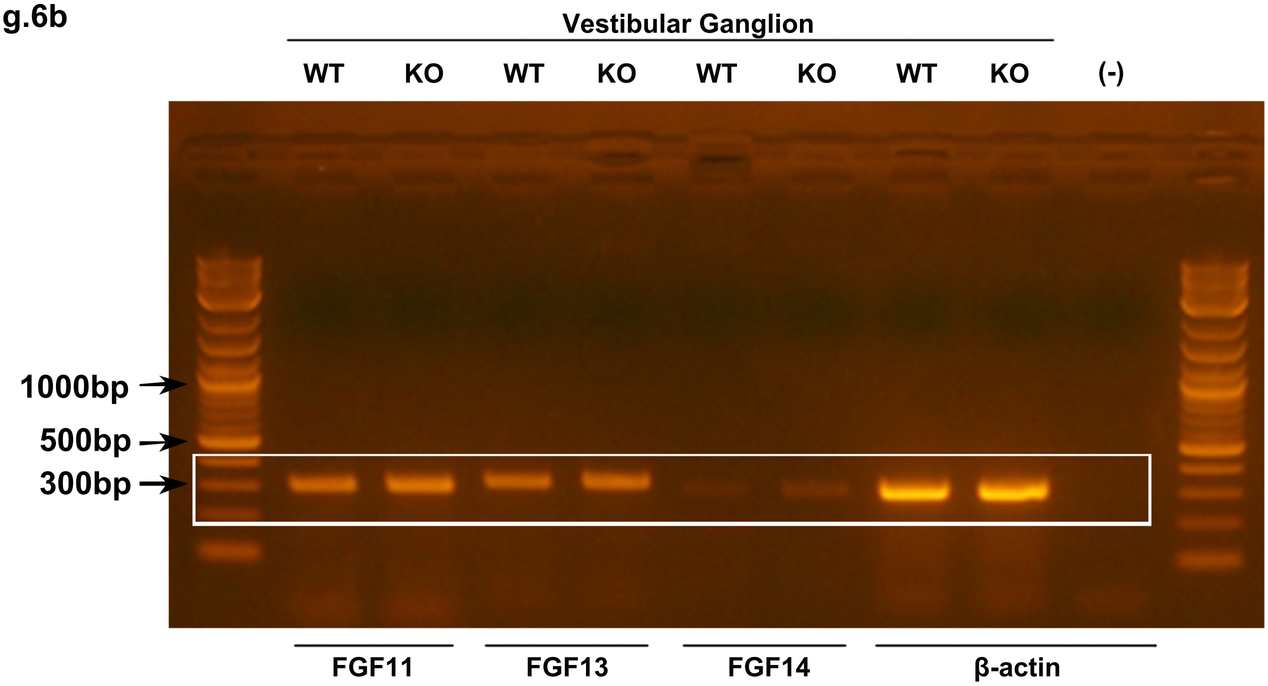

Figure. S-3.

Full-length gel for electrophoresis shown in Figure 6a and 6b.

Boxes indicate lanes that were used in each figure.
